# Supplementary material for: Resting-State Network Plasticity Induced by Music Therapy after Traumatic Brain Injury
Source: Neural Plast. 2021 Mar 8;2021:6682471. doi: 10.1155/2021/6682471 (PMC7964116; doi:10.1155/2021/6682471)
Supplement: Supplementary materials — The attached file of supplementary materials presents two tables with the results from the second-level analyses of the functional connectivity between and within networks and one figure showing the lesion overlap map. Table S1 shows the statistically significant results from the second-level analysis of functional connectivity between the nodes of the 4 resting-state networks of interest in this study (namely, frontoparietal, dorsal attention, salience, and default mode networks) with every other resting-state network included in the CONN toolbox. Table S2 shows the statistically significant results from the second-level analysis of functional connectivity within the nodes of the 4 resting-state networks of interest in this study (namely, frontoparietal, dorsal attention, salience, and default mode networks) included in the CONN toolbox. Figure S1 shows the lesion overlap map derived from 11 TBI patients with visible lesions. [file 6682471.f1.docx]

**Supplementary Material**

**Table S1.** **Between-network connectivity results.** This table shows the statistically significant results from the second-level analysis of functional connectivity between the nodes of the 4 resting-state networks of interest in this study (namely, Frontoparietal, Dorsal Attention, Salience and Default Mode networks) with every other resting-state network included in the CONN toolbox.

| **Pre- versus Post-Intervention Comparison (one-sided contrast)** | **T(22)** | **p-unc** | **p-FDR** |
| --- | --- | --- | --- |
| Frontoparietal-Lateral Prefrontal Cortex-Left AND Sensorimotor-Lateral-Right | 4.02 | 0.0003 | 0.0017 |
| Frontoparietal-Lateral Prefrontal Cortex-Left AND Dorsal Attention-Intraparietal Sulcus-  Right | 3.21 | 0.0020 | 0.0140 |
| Dorsal Attention -Intraparietal Sulcus-Right AND Visual-Occipital | 2.97 | 0.0035 | 0.0245 |
| Dorsal Attention -Intraparietal Sulcus-Right AND Visual-Lateral-Right | 2.61 | 0.0080 | 0.0281 |
| Dorsal Attention -Intraparietal Sulcus-Right AND Visual-Lateral-Left | 2.39 | 0.0130 | 0.0303 |
| **AB>BA and TP2>TP1 Interaction (one-sided contrast)** | **T(22)** | **p-unc** | **p-FDR** |
| Default Mode Network-Medial Prefrontal Cortex AND Sensorimotor-Superior | -3.20 | 0.0021 | 0.0128 |

*p-unc = p-uncorrected; p-FDR= False Discovery Rate-adjusted p at the source level.*

**Table S2. Within-network connectivity results.** This table shows the statistically significant results from the second-level analysis of functional connectivity within the nodes of the 4 resting-state networks of interest in this study (namely, Frontoparietal, Dorsal Attention, Salience and Default Mode networks) included in the CONN toolbox.

| **Pre- versus Post-Intervention Comparison (one-sided contrast)** | **T(22)** | **p-unc** | **p-FDR** |
| --- | --- | --- | --- |
| Frontoparietal-Lateral Prefrontal Cortex-Left AND Frontoparietal-Lateral Prefrontal Cortex-  Right | -2.58 | 0.0171 | 0.0494 |
| Frontoparietal-Lateral Prefrontal Cortex-Left AND Frontoparietal-Posterior Parietal Cortex-  Left | -2.28 | 0.0329 | 0.0494 |
| Salience-Supramarginal Gyrus-Left AND Salience-Supramarginal Gyrus-Right | -3.02 | 0.0063 | 0.0322 |
| Salience-Supramarginal Gyrus-Left AND Salience-Anterior Insula-Right | -2.79 | 0.0107 | 0.0322 |

*p-unc = p-uncorrected; p-FDR= False Discovery Rate-adjusted p at the source level.*


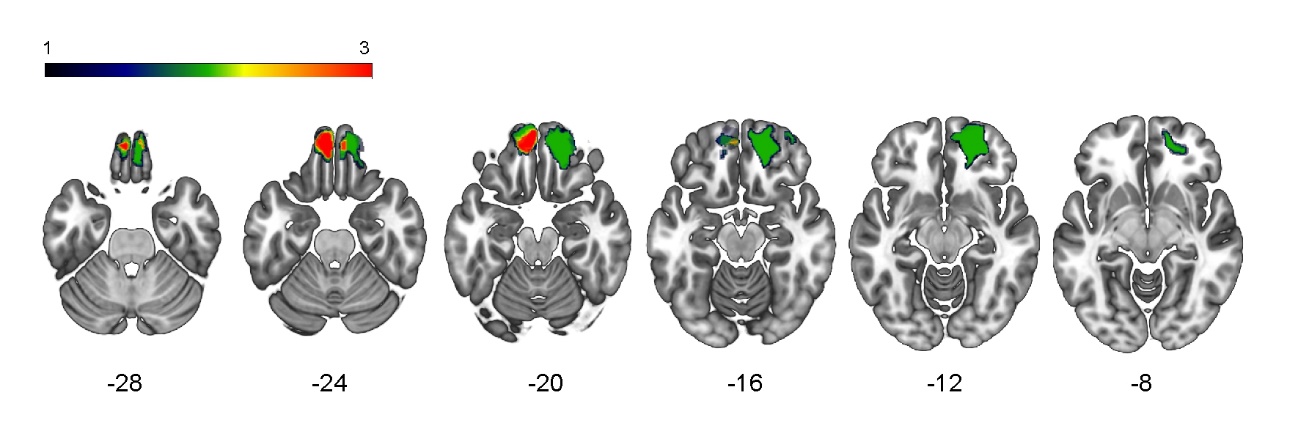


**Figure S1. Lesion overlap of 11 traumatic injury patients with visible lesions.** The colorbar indicates the number of patients with a lesion in each voxel (maximum 3 out of 11). Numbers below the axial slice denote the Z coordinate in mm.
